# Acute kidney injury after primary total hip arthroplasty: a risk multiplier for complication, mortality, and healthcare utilization

**DOI:** 10.1186/s13075-020-2116-3

**Published:** 2020-02-19

**Authors:** Jasvinder A. Singh, John D. Cleveland

**Affiliations:** 1 Birmingham Veterans Affairs (VA) Medical Center, Birmingham, 35233 AL USA; 2 Department of Medicine at the School of Medicine, University of Alabama at Birmingham, Faculty Office Tower 805B, 510 20th Street S, Birmingham, AL 35294 USA; 3 Division of Epidemiology at the School of Public Health, University of Alabama at Birmingham, Faculty Office Tower 805B, 510 20th Street S, Birmingham, AL 35294 USA

**Keywords:** Acute kidney injury, Total hip arthroplasty, THA, Healthcare utilization, Complications, Outcomes

## Abstract

**Objective:**

To assess whether acute kidney injury (AKI) is associated with more complications and higher healthcare utilization in people undergoing primary total hip arthroplasty (THA).

**Methods:**

Using a retrospective cohort study design, we performed multivariable-adjusted logistic regression of the 1998–2014 US National Inpatient Sample data to assess the association of AKI with complications (infection, transfusion, revision, and mortality) and healthcare utilization (total hospital charges, discharge to a rehabilitation facility, length of hospital stay) post-THA. We calculated the odds ratio (OR) and 95% confidence intervals (CI).

**Results:**

Adjusted for age, gender, race, income, underlying diagnosis, medical comorbidity, and the insurance payer, AKI in people who underwent primary THA was associated with significantly higher OR (95% CI) of (1) implant infection, 2.34 (95% CI, 1.87, 2.93); (2) transfusion, 2.46 (95% CI, 2.37, 2.56); (3) revision, 2.54 (95% CI, 2.16, 2.98); (4) death, 8.52 (95% CI, 7.47, 9.73); (5) total hospital charges above the median, 2.29 (95% CI, 1.99, 2.65); (6) discharge to a rehabilitation facility, 2.11 (95% CI, 2.02, 2.20); and (7) hospital stay > 3 days, 4.34 (95% CI, 4.16, 4.53).

**Conclusion:**

Quality improvement initiatives with optimization of the peri-operative care to reduce AKI and subsequently AKI-associated complications and healthcare utilization are needed. Mechanisms of AKI-associated post-THA complications need further examination.

## Background

Acute kidney injury (AKI) is an uncommon, but a clinically important post-surgical complication. AKI is a risk factor for the development of chronic kidney disease (CKD) and higher mortality [1]. Post-surgical AKI leads to higher adjusted healthcare costs (1.6-times) and longer hospital stays [2]. Thus, post-operative AKI leads to a major healthcare burden.

Total hip arthroplasty (THA) is the second most commonly performed arthroplasty in the US [3] with a growing demand due to associated pain relief and improvement in function. The incidence of AKI varied from 1% [4] to 8% [5] post-THA in two single-center studies, partially due to differences in the study sample (post-THA versus post-THA or post-TKA), the AKI definition (highest serum creatinine within 5 days post-operative vs. 7 days post-operative), and the time-trends with increased recognition over time (2004–2014 vs. 2013–2014). In a national US sample, the incidence of AKI in a combined sample of people who underwent knee or hip arthroplasty with an underlying diagnosis of osteoarthritis was 1.3% [6]; no estimates of AKI post-THA or associated complications, such as infection, were available. Complications after THA and knee arthroplasty differ. Most studies of AKI post-THA had significant limitations including single-center studies, small sample sizes, and unadjusted analyses.

In the absence of studies of incidence, correlates, and outcomes of AKI post-THA in the US with representative samples, our objective was to examine these aspects of AKI post-primary THA in a representative US sample. We hypothesized a priori that AKI would be associated with a higher rate of complications (infection, revision) and mortality (primary outcomes) after primary THA, and higher healthcare utilization and transfusion rates (secondary outcomes).

## Methods

### Data sources and study population

This retrospective cohort study used the data from the US National (formerly, Nationwide) Inpatient Sample (NIS), a 20% stratified sample of all discharges from US community hospitals, provided by the Agency for Healthcare Research and Quality (AHRQ) Healthcare Cost and Utilization Project (HCUP) [7]. The University of Alabama at Birmingham’s Institutional Review Board (IRB-120207004) approved this study and waived the need for informed consent. Study methods and results are reported in accordance with the Strengthening of Reporting in Observational studies in Epidemiology (STROBE) statement [8] (Additional file 1).

We included all hospitalizations for primary THA in the US from 1998 to 2014, identified by the presence of International Classification of Diseases, ninth revision, common modification (ICD-9-CM) code of 81.51, as the primary procedure code for hospitalization. The code-based algorithm is a validated approach to identify THA cohorts with positive predictive values of 98–99% [9]. The underlying diagnosis for THA was listed as the primary diagnosis ICD-9-CM code.

### Exposure of interest, study outcomes, and covariates

The exposure of interest, AKI, was identified by the presence of the ICD-9-CM code of 584.5, 584.6, 584.7, 584.8, or 584.9 as a non-primary (i.e., secondary) diagnosis. This code-based algorithm for AKI is also a validated approach with high positive predictive value of 98% [10].

Study outcomes were healthcare utilization and in-hospital post-operative complications. We assessed the length of hospital stay (above/below the median; 2.7 days rounded off to 3 days), the total hospital charges in US dollars (above/below median for each year), and the discharge disposition, i.e., to home vs. a rehabilitation facility which included short- or long-term care hospital, skilled nursing facility (SNF), intermediate care facility, or a certified nursing facility. Patients who left the hospital against medical advice were considered missing for discharge disposition. The key in-hospital post-operative complications include implant infection, transfusion, and THA revision and mortality.

Important covariates/potential confounders which included demographics (age, sex, race/ethnicity, income), the underlying diagnosis (osteoarthritis (OA), rheumatoid arthritis (RA), fracture, avascular necrosis of bone or other), comorbidity (Deyo-Charlson comorbidity index, a validated measure that included 17 comorbidities, based on the presence of ICD-9-CM codes), insurance payer (Medicare, Medicaid, private insurance, self-pay or other), and hospital characteristics were assessed. Hospital location/teaching status was categorized as rural, urban non-teaching, or urban teaching hospital. Hospital bed size was classified as small, medium, or large, using the NIS cutoffs that vary by the year. Hospital region was categorized as Northeast, Midwest, South, and West.

### Statistical analyses

We performed a separate multivariable-adjusted logistic regression models to assess each clinical (infection, transfusion, THA revision, and mortality) and healthcare utilization outcome (total hospital charges above/below the median, discharge to a rehabilitation facility, length of stay above/below the median of 3 days), adjusting for important potential predictors of post-THA complications and healthcare utilization and/or potential confounders of AKI outcomes [11]. We calculated the odds ratios (OR) and 95% confidence intervals (CI). We performed sensitivity analyses by additionally adjusting the main analyses for hospital characteristics. We used SAS 9.3 (Cary, NC) for all analyses.

## Results

Of the 4.1 million primary THAs, 61,077 (1.5%) developed AKI during the index primary THA hospitalization. AKI was associated with higher rate of complications and utilization in unadjusted analyses (Table 1).

**Table 1:** Demographic and other cohort characteristics for entire primary THA cohort including people without vs. with acute kidney injury (AKI) N (%), unless specified otherwise; *SE* standard error

	Entire Primary THA Cohort (*N* = 4,116,485)^a^	Primary THA	
		No Acute Kidney Injury (*N* = 4,055,407)^a^	Acute Kidney Injury (*N* = 61,077) ^a^
Age, Mean (SE); median	65.2 (0.04); 65.9	65.4 (0.04); 65.9	71.5 (0.12); 72.2
Age category, in years			
<50	449,642 (10.9%)	446,863 (11.0%)	2,779 (4.5%)
50-64	1,364,821 (33.2%)	1,351,472 (33.3%)	13,349 (21.9%)
65-79	1,732,014 (42.1%)	1,704,496 (42.0%)	27,518 (45.1%)
≥80	566,521 (13.8%)	549,094 (13.5%)	17,427 (28.5%)
Gender			
Female	2,330,188 (56.6%)	2,301,645 (56.8%)	28,543 (46.7%)
Male	1,776,722 (43.2%)	1,744,192 (43.0%)	32,530 (53.3%)
Race			
White	2,882,041 (70.0%)	2,838,722 (70.0%)	43,319 (70.9%)
Black	225,772 (5.5%)	219,916 (5.4%)	5,856 (9.6%)
Hispanic	104,385 (2.5%)	102,556 (2.5%)	1,829 (3.0%)
Other/Missing	904,234 (22.0%)	894,166 (22.0%)	10,068 (16.5%)
Primary Diagnosis			
Rheumatoid arthritis (RA)	29,173 (0.7%)	29,016 (0.7%)	157 (0.3%)
Avascular bone necrosis	285,623 (6.9%)	281,334 (6.9%)	4,289 (7.0%)
Osteoarthritis (OA)	3,447,224 (83.7%)	3,405,538 (84.0%)	41,686 (68.3%)
Other	354,307 (8.6%)	339,372 (8.4%)	14,935 (24.5%)
Fracture	117 (0.003%)	107 (0.003%)	10 (0.02%)
Deyo-Charlson Score			
0	2,193,575 (53.3%)	2,181,133 (53.8%)	12,442 (20.4%)
1	926,286 (22.5%)	913,996 (22.5%)	12,290 (20.1%)
≥2	996,623 (24.2%)	960,278 (23.7%)	36,345 (59.5%)
Insurance			
Medicaid	138,809 (3.4%)	136,558 (3.4%)	2,251 (3.7%)
Medicare	2,234,675 (54.3%)	2,190,406 (54.0%)	44,269 (72.5%)
Other	102,277 (2.5%)	101,017 (2.5%)	1,260 (2.1%)
Private	1,600,829 (38.9%)	1,588,027 (39.2%)	12,802 (21.0%)
Self	32,307 (0.8%)	31,939 (0.8%)	368 (0.6%)
Income Category^b^			
0-25^th^ percentile	653,243 (15.9%)	639,522 (15.8%)	13,721 (22.5%)
25-50^th^ percentile	1,009,678 (24.5%)	994,108 (24.5%)	15,570 (25.5%)
50-75^th^ percentile	1,086,953 (26.4%)	1,071,309 (26.4%)	15,644 (25.6%)
75-100^th^ percentile	1,285,854 (31.2%)	1,270,801 (31.3%)	15,053 (24.6%)
Hospital Location/Teaching			
Rural	444,188 (10.8%)	439,410 (10.8%)	4,778 (7.8%)
Urban	1,722,390 (41.8%)	1,696,986 (41.8%)	25,404 (41.6%)
Urban Teaching	1,939,988 (47.1%)	1,909,531 (47.1%)	30,457 (49.9%)
Hospital Bed size			
Small	685,208 (16.6%)	676,555 (16.7%)	8,653 (14.2%)
Medium	1,037,562 (25.2%)	1,021,335 (25.2%)	16,227 (26.6%)
Large	2,383,797 (57.9%)	2,348,038 (57.9%)	35,759 (58.5%)
Hospital Region			
Northeast	818,699 (19.9%)	807,610 (19.9%)	11,089 (18.2%)
Midwest	1,089,883 (26.5%)	1,073,918 (26.5%)	15,965 (26.1%)
South	1,358,857 (33.0%)	1,334,688 (32.9%)	24,169 (39.6%)
West	849,046 (20.6%)	839,192 (20.7%)	9,854 (16.1%)

^a^The national estimates were based on the following from the 20% NIS sample: Entire cohort (*N* = 855,634); no AKI (*N* = 843,044); AKI (*N* = 12,590)

^b^Income quartiles were defined as follows: (1) from 2003, ZIPINC_QRTL variable is available, which was used; (2) 1998-2002: another categorical variable (ZIPINC) indicated the median household income of the patient's residential ZIP Code, based on the 1999 demographics. The categories were defined so that the maximum for category 1 ($25,000) was approximately 150% of the 1999 poverty level and the boundary between the second and third categories ($35,000) was approximately the national median household income. As an example, for 2014, Q1 (0-25^th^ percentile) was $1 - 39,999, Q2 was $40,000 - 50,999, Q3 was $51,000 - 65,999; and Q4 was $66,000+. In 1998, Q1 was $1 - 28,999, Q2 (25-50^th^ percentile) was $29,000 - 36,999, Q3 (50-75^th^ percentile) was $37,000 - 49,999; and Q4 (75-100^th^ percentile) was $50,000+

Unadjusted estimates were higher for each in-hospital complication (infection, transfusion, revision) by 2-fold and mortality (20-fold) in people with AKI compared to those without AKI (Table 2). Unadjusted total hospital charges, proportion discharged to a rehabilitation facility, and the length of hospital stay were higher in people with AKI (Table 2).

**Table 2:** Unadjusted estimates for in-hospital complications and healthcare utilization outcomes for the index hospitalization for primary THA for the entire cohort and by the presence/absence of AKI

	Entire Primary THA Cohort (*N* = 4,116,485)^a^	Primary THA	
		No Acute Kidney Injury (*N* = 4,055,407)^a^	Acute Kidney Injury (*N* = 61,077) ^a^
In-hospital Complications^b^			
Infection	7,592 (0.2%)	7,173 (0.2%)	419 (0.7%)
Revision	17,931 (0.4%)	17,252 (0.4%)	679 (1.1%)
Transfusion	937,803 (22.8%)	912,311 (22.5%)	25,492 (41.7%)
Death	8,889 (0.2%)	6,538 (0.2%)	2,351 (3.8%)
Healthcare Utilization Outcomes			
Discharge Status			
Home	2,448,107 (59.5%)	2,427,284 (59.9%)	20,823 (34.1%)
Inpatient facility^c^	1,649,102 (40.1%)	1,611,362 (39.7%)	37,740 (61.8%)
Length of hospital stay, Mean (SE); median	3.71 (0.01); 2.74	3.68 (0.01); 2.74	7.15 (0.07); 4.49
Length of hospital stay categories			
≤3 days	2,499,883 (60.7%)	2,483,373 (61.2%)	16,510 (27.0%)
>3 days	1,616,602 (39.3%)	1,572,034 (38.8%)	44,568 (73.0%)
Total Hospital Charges, in U.S. $, Mean (SE); Median	44,635 (268); 37,658	44,313 (266); 37,537	81,202 (911); 61,686
1998-2000	23,556 (275); 20,858	23,455 (274); 20,838	56,412 (3,039); 39,461
2001-2002	29,210 (431); 25,432	29,044 (427); 25,398	63,621 (3,521); 41,480
2003-2004	36,086 (598); 31,359	35,805 (597); 31,289	72,805 (3,355); 49,318
2005-2006	40,678 (623); 35,383	40,369 (621); 35,290	70,439 (2,308); 50,555
2007-2008	47,216 (787); 41,423	46,764 (777); 41,291	77,075 (2,668); 55,301
2009-2010	49,918 (1,062); 43,304	49,305 (1,054); 43,022	77,483 (1,894); 60,193
2011-2012	56,395 (848); 48,917	55,681 (833); 48,600	86,252 (2,211); 67,974
2013-2014	58,964 (532); 51,157	58,230 (528); 50,851	90,271 (1,775); 69,643

N (%), unless specified otherwise; *SE* standard error

^a^The national estimates were based on the following from the 20% NIS sample: Entire cohort (*N* = 855,634); no AKI (*N* = 843,044); AKI (*N* = 12,590)

^b^In-hospital complications during the index hospitalization for primary THA

^c^Inpatient facility included short- or long-term care hospital, skilled nursing facility (SNF), intermediate care facility, or a certified nursing facility

Adjusted for age, gender, race, income, underlying diagnosis, medical comorbidity, and insurance payer, AKI was associated with significantly higher OR of poorer outcomes: (1) infection, 2.34 (95% CI, 1.87, 2.93); (2) transfusion, 2.46 (95% CI, 2.37, 2.56); (3) revision, 2.54 (95% CI, 2.16, 2.98); (4) death, 8.52 (95% CI, 7.47, 9.73); (5) total hospital charges above the median, 2.94 (95% CI, 2.79, 3.03); (6) discharge to a rehabilitation facility, 2.11 (95% CI, 2.02, 2.20); and (7) the length of hospital stay > 3 days, 4.34 (95% CI, 4.16, 4.53) (Table 3). Sensitivity analyses that additionally adjusted the main model for hospital characteristics confirmed the main findings with minimal/no attenuation (Table 3).

**Table 3:** Multivariable-adjusted association of AKI with healthcare utilization outcomes and in-hospital complications for index hospitalization for primary THA in the main model and sensitivity analysis

	Main Model^a^	Sensitivity analyses^b^: Main Model + hospital characteristics
	OR (95% CI)	OR (95% CI)
Total hospital charge above the median ^c^	**2.29 (1.99, 2.65)**	**2.25 (1.95, 2.60)**
Discharge to an inpatient/rehabilitation facility^d^	**2.11 (2.02, 2.20)**	**2.15 (2.06, 2.25)**
Length of hospital stay more than median (>3 days)^e^	**4.34 (4.16, 4.53)**	**4.39 (4.20, 4.58)**
In-hospital complications^f^		
Infection	**2.34 (1.87, 2.93)**	**2.33 (1.87, 2.91)**
Transfusion	**2.46 (2.37, 2.56)**	**2.52 (2.42, 2.61)**
Revision	**2.54 (2.16, 2.98)**	**2.56 (2.17, 3.01)**
Death	**8.52 (7.47, 9.73)**	**8.54 (7.48, 9.76)**

BOLD indicates significant odds ratio

^a^Main model was adjusted for socio-demographics (age, race/ethnicity, gender, income), the Deyo-Charlson comorbidity index, insurance payer and the underlying diagnosis for primary THA

^b^Sensitivity analyses adjusted each main model additionally for hospital characteristics including hospital location/teaching status, hospital region and hospital bed size

^c^Median hospital charges were calculated for each year

^d^Inpatient/rehabilitation facility included short- or long-term care hospital, skilled nursing facility (SNF), intermediate care facility, or a certified nursing facility

^e^Median length of hospital stay was 2.74 days, rounded off to 3 days

^f^In-hospital complications

## Discussion

In this national study of > 4 million primary THAs using the 1998–2014 US NIS, we found the diagnosis of AKI in 1.5% of all primary THA hospitalizations, similar to the incidence of 1.3–1.5% reported previously in a combined knee/hip cohort or from a single-center [4, 6]. To our knowledge, this is the first study to provide data on AKI post-THA based on the redesign of NIS in 2012 [7] intended to improve the national estimates. Several findings merit further discussions.

The absolute increase in post-primary-THA complications with AKI ranged 0.5% for implant infection to 19% for transfusion. This finding is clinically important, given that the rate of complications and mortality rates in those without AKI were 0.2–0.4% except that for transfusion (22.5%). The multivariable-adjusted relative risk of in-hospital complications was 2.3–2.5 times. Most primary THAs are elective procedures. Our findings identify AKI as an important risk factor for complications post-primary THA.

Potential mechanisms of post-operative AKI involve hemodynamic, inflammatory, and nephrotoxic factors [12], including angiotensin-converting enzyme inhibitor/angiotensin receptor blockers (ACE inhibitors/ARBs) and non-steroidal anti-inflammatory drugs (NSAIDs), which can be associated with added toxicity when used concomitantly with each other. American Society of Anesthesiologist (ASA) score, cardiovascular disease, diabetes, creatinine > 2 mg/dl, and high-risk surgery were significant predictors of post-operative AKI [13]. In THA populations, NSAIDs were either associated with 2.7-times higher risk of AKI [14] or not associated with AKI post-THA [4, 5]. A recent review identified obesity, metabolic syndrome, perioperative antibiotics, antihypertensives medications, and antibiotic-impregnated cement spacers as risk factors for AKI post-knee arthroplasty [15]. These findings may likely applicable to post-THA AKI. Patient education, careful avoidance of nephrotoxic medications in peri-operative including those leading to hypotension, maintenance of adequate intravascular volume, and effective comorbidity management (cardiac, vascular, pulmonary, renal, diabetes, etc.) may also help in reducing the risk of post-THA AKI. Studies of quality initiatives of these interventions (alone or in combination) are needed to generate evidence to support these approaches in clinical practice.

AKI during index primary THA hospitalization was independently associated with higher healthcare utilization in multivariable-adjusted analyses. Our study found a 3.5-day longer hospital stay and $37,000 excess mean hospital charges (unadjusted) and 8.5-fold higher adjusted mortality associated with AKI post-THA, extends findings from a single-center study [16] and from a study that combined primary and revision knee/hip arthroplasty performed only for osteoarthritis [6]. AKI was also associated with a longer hospital stay and 2.1-times higher odds of discharge to a rehabilitation facility.

Study strengths include the use of a national, representative sample and adjustment for several potential confounders. Our study findings must be interpreted carefully considering the study limitations. The NIS excludes federal facilities such as military hospitals and Veterans Affairs medical centers, and therefore, the findings are not generalizable to these cohorts. The NIS counts hospitalizations and not procedures, i.e., bilateral procedures are missed and counted as single procedures. Since simultaneous bilateral THAs are uncommon (< 1%), this is unlikely to have affected our estimates greatly. We examined hospital charges, not actual hospital costs or what was reimbursed, which is usually lower than the charges. Misclassification bias is possible, since we used ICD-9-CM codes to identify both AKI and TKA, despite a high validity of codes for both conditions [9, 10]. Longitudinal follow-up is desirable for post-THA outcomes; however, NIS data do not lend themselves to such analyses. Another limitation is the lack of a published protocol for this observational study, although we specified more complications, and higher healthcare utilization and mortality rate associated with AKI post-THA a priori.

## Conclusions

In conclusion, in a national cohort of patients undergoing primary THA, AKI occurred in 1.5% of all hospitalizations. AKI in patients who underwent primary THA was independently associated with each of the four post-operative in-hospital complications in adjusted analyses, including mortality. AKI was also associated with higher healthcare utilization post-THA in adjusted analyses. Quality initiatives that further improve the care pathways for patients undergoing primary THA may reduce the risk of AKI, and associated complications and excess healthcare utilization. Research studies need to explore the mechanisms of AKI in patients undergoing primary THA and examine interventions that target modifiable risk factors for AKI.

## Supplementary information

**Additional file 1.** Mapping of the various components in our manuscript of the STROBE Statement checklist of items for reports of observational studies.

## Data Availability

We are ready to share the data with colleagues, after obtaining appropriate permissions from the University of Alabama at Birmingham (UAB) ethics board and done in compliance the UAB patient privacy rules and data sharing policies.

## Abbreviations

AKI: Acute kidney injury
THA: Total hip arthroplasty
NIS: National (formerly, Nationwide) Inpatient Sample
AHRQ: Agency for Healthcare Research and Quality
HCUP: Healthcare Cost and Utilization Project
ICD-9-CM: International Classification of Diseases, ninth revision, common modification
US: United States
OR: Odds ratio
CI: Confidence intervals
SE: Standard error of the mean
